# Effects of αβ-amirona on inflammation in topical edema ear model in mice

**DOI:** 10.1186/1753-6561-8-S4-P35

**Published:** 2014-10-01

**Authors:** Natália Ferreira, Rafael Monteiro, Geane Lourenço

**Affiliations:** 1 Curso de Biotecnologia - UFAM, Manaus, Brasil; 2 Laboratório de Farmacologia - ICB- UFAM, Manaus, Brasil

## Introduction

*In vivo *models of inflammation are definitely the best tools to study the biological role, because no other approach will allow the simultaneous investigation of cells and mediators that interact during the inflammatory process. Several reports in the literature have shown the efficacy of α, β-amyrin, the main component of the resin plant of the genus Protium in studies with in vitro models of inflammation. Another component of this resin with anti-inflammatory activity is the αβ-amirona, but this is still less studied.

Objective: To evaluate the topical anti-inflammatory activity of the compound isolated αβ-amirona in models of skin inflammation in mice.

## Methods

We used 30 male mice of the Balb-c strain weighing 30-35g were divided into 6 groups (control phenol, treated with the extract at concentrations of 0.6 mg/ear, 0.3 mg/ear, 0.1 mg/ear and control ears treated with dexamethasone). Edema was induced by topical application of 10% phenol in acetone (20 mL/ear) on the right ear of all groups. The extract (0.6, 0.3 and 0.1 mg/ear) or dexamethasone (0.1 mg/ear) was used as positive control, were applied directly after phenol. The left ear of each animal was used as a control for evaluation of edema. One hour after treatment, the animals were anesthetized with thiopental 80mg/kg and sacrificed by intracardiac injection of potassium chloride 3M, 5mm circles were taken from their ears with the aid of a punsh biopsy and weighed on an analytical balance. Evaluation edema was taken as the difference in weight of the tissue samples.

## Results

The group of animals that received topical treatment with solution of αβ-amirona exhibited a decrease in edema induced by phenol when compared to the control group. However, the positive control group that was treated with dexamethasone showed a sharper decrease edema.

## Conclusions

The compound herbal αβ-amirona showed topical anti-inflammatory action, in the model of ear edema induced by phenol in mice, but at a lower power action of dexamethasone.

## References

[B1] Aller MA Arias JL Sánchez-Patán F Arias J The inflammatory response: an efficient way of life Medical Science Monitor 2006 12 225 234 17006415

[B2] Calixto JB Campos MM Otuki MF Santos AR Antiinflammatory compounds of plant origin. Part II. modulation of proinflammatory cytokines, chemokines and adhesion molecules Planta Medica 2004 70 93 103 14994184 10.1055/s-2004-815483

[B3] De Lima RA Velho LMLS Faria LIL Indicadores bibliométricos de cooperação científica internacional em bioprospecção Perspectivas em Ciências da Informação 2007 12 1 50 64

[B4] Gàbor M Mouse Ear Inflammation Models and their Pharmacological Applications Budapeste: Akadémiai Kiadó 2000

[B5] Schultes RE Raffauf RF The healing forest medical and toxic plants of the Northwest Amazonia 1990 Portland, OR: Dioscorides Press

